# Health beliefs and breast self-examination practices among female health science students in Hungary: a health belief model perspective

**DOI:** 10.3389/fpubh.2025.1681802

**Published:** 2025-11-05

**Authors:** Sára Garai, Johanna Törzsökné Márton, Dávid Sipos, Melinda Csima

**Affiliations:** ^1^Doctoral School of Health Sciences, Faculty of Health Sciences, University of Pécs, Pécs, Hungary; ^2^Department of Medical Imaging, Faculty of Health Sciences, University of Pécs, Pécs, Hungary; ^3^Institute of Education, Hungarian University of Agriculture and Life Sciences, Kaposvár, Hungary

**Keywords:** breast self-examination, breast cancer awareness, health belief model, health education, health-conscious behavior, female university students

## Abstract

**Background/objectives:**

Breast cancer remains one of the leading health concerns for women worldwide, while methods promoting early detection, such as breast self-examination (BSE), are still insufficiently integrated into preventive practices. The aim of this study was to explore health beliefs related to BSE and the factors influencing them among female students of the Faculty of Health Sciences at the University of Pécs, using the Health Belief Model (HBM).

**Methods:**

A quantitative, cross-sectional study was conducted with the participation of 251 students, who assessed perceived susceptibility, severity, benefits, barriers, self-efficacy, and health motivation regarding BSE using the Champion Health Belief Model Scale (CHMBS).

**Results:**

The study found that 50.2% of students performed BSE regularly, 41.8% occasionally, and 8% never. A family history of breast cancer, especially cases in-volving grandmothers, was significantly associated with the practice of breast self-examination (χ^2^ = 4.437, *p* = 0.035). Self-efficacy was the strongest predictor (OR = 1.67; *p* < 0.001), while perceived barriers (OR = 0.68; *p* = 0.012), perceived severity (OR = 1.32; *p* = 0.003), and BSE knowledge (OR = 1.23; *p* = 0.035) also influenced engagement. Students with higher self-efficacy (χ^2^ = 12.875, *p* = 0.012) and better knowledge of breast cancer prevention were more likely to practice BSE. Information from gynecologists and family played a crucial role in BSE adherence.

**Conclusion:**

This study offers a new perspective for professionals by emphasizing the need for targeted health education programs that focus on strengthening self-efficacy and reducing perceived barriers. Our findings are of significant public health importance, as they support the integration of structured BSE education into university curricula, thereby fostering the development of health-conscious behaviors that promote early detection among young women.

## 1 Introduction

Breast cancer represents the most significant global cancer burden among women, being the most commonly diagnosed cancer and the leading cause of cancer-related deaths in the female population. It ranks first in incidence in 157 countries and first in mortality in 112 countries, accounting globally for nearly one in four cancer cases and one in six cancer deaths among women (1). In terms of overall cancer types, female breast cancer is the second most common malignancy worldwide, with an estimated 2.3 million new cases diagnosed in 2022, representing 11.6% of all cancer diagnoses (1). Despite this, breast cancer remains the fourth leading cause of cancer-related death overall, accounting for approximately 666,000 deaths annually and 6.9% of total cancer mortality (1). The incidence and mortality rates of the disease vary by region and are influenced by a society’s economic development, access to healthcare systems, and the effectiveness of screening programs (2). Considering morbidity and mortality indicators, the WHO Global Breast Cancer Initiative (GBCI) has set a goal to reduce breast cancer mortality by 2.5% annually worldwide, thereby creating an opportunity to save 2.5 million lives (3).

In Hungary, breast cancer is among the most common malignant diseases affecting women. According to data from the National Cancer Registry, more than 8,000 new cases have been reported annually since 2015 (4). Based on 2021 data, the avoidable breast cancer mortality rate was 24 per 100,000 women, which is 29% higher than the European Union average (5). Hungary currently has the third-highest overall breast cancer mortality rate in Europe, approaching levels seen in more developed countries - despite the availability of organized screening programs since 2002 (6).

Breast cancer is a complex, multifactorial disease influenced by various obstetric, gynecological, reproductive, and endocrine factors. The underlying causes of breast cancer encompass a wide range of risk factors, including a personal or family history of the disease, obesity, tall stature, smoking, alcohol consumption, early menarche, late menopause, a sedentary lifestyle, nulliparity, and the use of hormone replacement therapy (7). Age is considered an independent and significant risk factor, and the cumulative number of menstrual cycles is also crucial, highlighting the key role of endogenous estrogen in the disease’s pathogenesis. Additionally, research has confirmed that women who have their first childbirth after the age of 30 face a twofold increase in the risk of developing breast cancer (8). Current breast cancer prevention strategies include lifestyle modifications (such as avoiding smoking, maintaining a healthy weight, exercising, breastfeeding, eating a balanced diet, and limiting alcohol), chemoprevention for high-risk women, and prophylactic surgeries like mastectomy or oophorectomy in those with high-risk gene mutations. Emerging approaches focus on precision medicine and the role of the immune system and tumor microenvironment, enabling more personalized prevention (9).

Breast self-examination (BSE) was initially introduced as an intuitive, inexpensive, non-invasive, and universally accessible method for the early detection of breast neoplasms. As a cost-effective screening tool, BSE enables the timely identification of suspicious changes in breast tissue, thereby facilitating the early diagnosis of breast cancer (10, 11). The American Cancer Society (ACS) emphasizes the importance of breast awareness and recommends that women be familiar with the normal condition of their breasts and report any changes to healthcare professionals (12). However, the effectiveness of BSE in predicting diagnosis and reducing mortality rates remains a topic of debate, as evidence supporting its impact is still limited (13, 14).

Although exact data on the BSE practices of female university students in Hungary are not available, domestic studies conducted among the adult population indicate significant gaps in both knowledge and practical application. Previous research findings suggest that while the majority of respondents have heard of BSE, only a small proportion possess adequate theoretical knowledge and practical skills for its correct execution (15–17). Several studies indicate that regular breast self-examination practice remains low even among healthcare students and professionals. Only 32.9% of Croatian health science students and 45.4% of Greek midwifery students reported performing BSE monthly (18, 19). In many countries, it can be observed that although most women are aware of the importance of BSE, only a small proportion perform it regularly. For example, only 18% of female university students in Saudi Arabia reported performing BSE; in Bangladesh, only 7.4% practiced it monthly; and in Malaysia, 27.5% reported ever performing BSE (20).

Individual beliefs and perceptions play a crucial role in health behavior, with the Health Belief Model (HBM) serving as a socio-psychological framework to explain these patterns. The HBM examines factors influencing health-related decision-making, such as perceived susceptibility, expected severity of illness, benefits of preventive interventions, and potential barriers. It is widely applied in predicting and influencing various preventive behaviors, including breast cancer screening practices (21–24).

The model centers on two key dimensions of individual health behavior: perceived threat and behavioral evaluation. Perceived threat refers to a person’s beliefs about their susceptibility to a health condition and the perceived severity of its potential consequences. Behavioral evaluation involves the perceived benefits of engaging in the recommended health behavior, as well as the perceived barriers that may hinder its adoption. Additionally, the model incorporates general health motivation, reflecting an individual’s overall inclination to maintain and protect their health (25). This study is based exclusively on the Health Belief Model, which serves as the sole theoretical framework for analyzing preventive health behavior, particularly breast self-examination (BSE).

Several international studies have demonstrated the applicability of the HBM in understanding BSE practices, although their findings vary. For instance, research from Turkey found that perceived benefits and self-efficacy were positively associated with BSE practice (26), while other studies did not observe significant relationships (27, 28). In contrast, studies from Iran and Indonesia suggest that both perceived benefits and self-efficacy are key predictors of BSE behavior (29, 30). Research from Mexico and Yemen also highlighted the influence of benefit perception on screening behavior, although the strength of associations varied (31, 32).

The HBM’s relevance is further underscored by its operationalization through validated instruments. Champion (33) was the first to apply the HBM framework specifically to explore women’s beliefs about breast cancer and breast screening behaviors, including BSE and mammography. As a result, the Champion’s Health Belief Model Scale (CHBMS) was developed (34, 35), which has since become one of the most widely used tools to predict screening behaviors. The scale has been translated into several languages and adapted to various cultural contexts, making it a robust instrument for cross-cultural studies.

Given that the HBM captures key individual cognitive factors influencing preventive behavior, it provides a strong theoretical foundation for the present study. This model is particularly suitable for examining how female university students in Hungary perceive their breast cancer risk and make decisions regarding self-examination. Applying this model allows for the assessment of critical variables such as perceived susceptibility, perceived severity, benefits, barriers, and self-efficacy, which are essential for designing effective health education and promotion strategies.

Focusing on university students is particularly justified, as educational institutions play a crucial role in shaping health behavior. The university environment offers an ideal context for young individuals to learn about breast cancer risk factors, screening options, and the importance of health awareness. At this critical stage in life, long-term health behaviors are established, and health-related autonomy increases. However, studies indicate that knowledge and practice of breast self-examination (BSE) remain limited among young women, particularly in low- and middle-income countries (36, 37). This highlights the importance of targeted educational interventions during higher education. Health science students represent a particularly important subgroup, as they are not only potential future participants in screening programs but also future health educators. Their beliefs and behaviors can influence both their immediate social environment and, later, public health outcomes through their professional roles. Therefore, understanding their health beliefs and preventive practices is essential for supporting both individual and population-level health promotion. Although numerous international studies have investigated breast self-examination practices and related health beliefs, often within the framework of the HBM, no comprehensive research has been conducted in Hungary among female university students, and the HBM has not yet been applied to this population. Addressing this gap is essential, as the university setting offers a valuable opportunity to foster awareness and preventive health behaviors. Furthermore, no known survey has specifically focused on this age group to map their cognitive health beliefs, making our study a significant contribution in this field. The aim of our research was to explore knowledge and practical application of BSE, as well as to examine the influence of socio-demographic factors and key dimensions of the HBM, including perceived susceptibility, severity, benefits, barriers, self-efficacy, and health motivation.

This study aims to explore BSE-related knowledge and practices, and to assess the role of socio-demographic variables and key HBM constructs in shaping students’ health behavior. While the single-site design and use of a non-random sample restrict the extent to which the findings can be generalized, they may nonetheless provide meaningful preliminary insights for the development of targeted health education and promotion strategies in university settings.

Although breast self-examination (BSE) is not routinely recommended for all young women, it is still considered useful for familiarizing individuals with their own bodies from early adulthood. In this context, focusing on health science students is particularly relevant, as they represent future health professionals who may play an important role in disseminating knowledge and promoting preventive practices. Their engagement with BSE can therefore be seen as both a personal health behavior and a potential model for wider health promotion.

## 2 Materials and methods

### 2.1 Study population, sampling criteria, and data collection

A cross-sectional, descriptive study was conducted in September 2024 at the Faculty of Health Sciences, University of Pécs, utilizing a non-random, voluntary sampling method. The study population consisted of female undergraduate students enrolled at the Faculty, regardless of their academic discipline or year of study. Female students were intentionally selected, as breast self-examination (BSE) is a gender-specific behavior. The only exclusion criterion was a previous or current diagnosis of breast cancer. No further exclusion criteria were applied in order to ensure a broad and diverse sample. A total of 252 responses were collected, with one excluded due to non-eligibility, resulting in a final sample size of 251 participants. The response rate was 100%.

Data were collected prior to the commencement of an elective university course on breast cancer through a self-administered online questionnaire. Online informed consent was obtained from all participants prior to inclusion. Participation was voluntary, and no personal identifiers were collected to ensure confidentiality and anonymity. The questionnaire was completed entirely online, and all data were handled exclusively by the research team.

This study was approved by the Scientific and Research Ethics Committee of the Health Science Council (protocol number: BM/23045–3/2024; approval date: 15 October 2024) and was conducted in accordance with the ethical principles outlined in the Declaration of Helsinki.

### 2.2 Measures

The structured questionnaire comprised two sections. The first section collected demographic information, including age, marital status, type of residence, study mode (full-time or part-time), and family history of breast cancer. It also assessed participants’ knowledge and practice of BSE, the sources of information they had encountered, and the frequency and method of practicing BSE.

The second section consisted of the Champion Health Belief Model Scale (CHBMS), which evaluated health beliefs related to BSE across eight dimensions using a five-point Likert scale (1 = strongly disagree to 5 = strongly agree). The CHBMS was translated into Hungarian using a forward–backward translation process. Content validity was verified by two public health researchers and one breast cancer specialist. A pilot study with 30 participants was conducted to test reliability and clarity, after which minor linguistic refinements were made. Internal consistency was confirmed using Cronbach’s alpha values for each subscale (see Table 1).

**Table 1:** Reliability and versions of health belief model subscales.

Subscale	Number of items	Version	Cronbach’s alpha
Perceived susceptibility	3	1999	0.82
Perceived severity	7	1993	0.80
Perceived benefits of BSE	6	1993	0.85
Perceived barriers to BSE	9	1997	0.78
Self-efficacy for BSE	10	1997	0.89
Health motivation	7	1993	0.75
Perceived benefits of mammography	2	1993	0.93
Perceived barriers to mammography	5	1999	0.81

### 2.3 Statistical analysis

The required sample size was calculated using G*Power software (version 3.1.9.4), based on the tests planned in the study. An *a priori* power analysis using the t-test family indicated that a minimum of 57 participants per group (114 in total) would be required to detect statistically significant differences (effect size *d* = 0.53; *α* = 0.05; power = 0.80; allocation ratio N1/N2 = 1). Further calculations showed that a minimum of 63 participants were needed for logistic regression (medium effect size) and 87 for chi-square tests (*w* = 0.3). The final sample of 251 exceeded these thresholds, ensuring sufficient power for the statistical analyses.

## 3 Results

The descriptive statistics are presented in Table 2. Of the 251 participants, 50.2% reported performing BSE regularly, 41.8% occasionally, and 8.0% never. The mean age of the students in the study was 23.93 years (SD = 7.76), with a median age of 21 years. The age range of participants spanned from 18 to 53 years. Among the participants, 47.0% were in a relationship, 39.4% were single, and 13.5% were married. The majority of respondents resided in a town (61.4%), while 31.9% lived in a village or rural area, and only 6.7% were residents of the capital city. Regarding study mode, the majority of students were enrolled in full-time education (70.5%), while 29.5% were part-time students.

**Table 2:** Characteristics of the respondents (N = total sample size; n = number of respondents; % = proportion within the given category).

Variable *N* = 251	Performs BSE (n) *N* = 178	Performs BSE (%)	Does not perform BSE *N* = 73	Does not perform BSE (%)	X2/[df]	*p*-value	Cramer’s V
Age group						**0.040**	0.160
18–29	143	67.7	68	32.2	6.413 [2]		
30–39	18	85.7	3	14.3			
40–53	17	89.5	2	10.5			
Marital status					6.836 [2]	**0.033**	0.165
Single	64	64.7	35	35.3			
In a relationship	84	71.2	34	28.8			
Married	30	88.3	4	11.7			
Place of residence					11.663 [2]	**0.003**	0.216
Village	61	76.3	19	23.7			
Town	111	72.1	43	27.9			
Capital	6	35.3	11	64.7			
Study mode					7.109 [1]	**0.008**	0.168
Full-time	122	66.3	62	33.7			
Part-time	56	83.6	11	16.4			
Trust in healthcare system and doctors					11.147 [2]	**0.004**	0.215
Low	40	58.8	28	41.2			
Moderate	113	72.4	43	27.6			
High	25	92.6	2	7.4			
Family history of breast cancer: yes							
Grandmother	40	83.3	8	16.7	4.437 [1]	**0.035**	0.133
Mother	16	66.7	8	33.3	0.232 [1]	0.630	0.030
Aunt	8	72.7	3	27.3	0.018 [1]	0.892	0.091
Presence of children					5.578 [1]	**0.018**	0.149
Yes	33	18.6	144	81.4			
No	5	6.8	68	93.2			
* Sources of information on BSE							
Television	24	13.5	3	4.1	4.738 [1]	**0.029**	0.137
Books, newspapers, magazines	16	9.0	4	5.5	0.869 [1]	0.351	0.059
Internet	65	36.5	27	37.0	0.005 [1]	0.944	0.004
Social media	46	25.8	13	17.8	1.859 [1]	0.173	0.086
Flyers, posters	19	10.7	3	4.1	2.790 [1]	0.095	0.105
Family member	99	55.6	51	69.9	4.368 [1]	**0.037**	0.132
Friends, colleagues	136	76.4	63	86.3	3.087 [1]	0.079	0.111
Healthcare professionals	113	63.5	62	84.9	11.281 [1]	**<0.001**	0.212
General practitioners	146	82.0	71	97.3	10.264 [1]	**<0.001**	0.202
Gynecologist	96	70.9	73	29.1	15.848 [1]	**<0.001**	0.251
School	95	53.4	47	64.4	2.556 [1]	0.110	0.101
*Sources for learning proper BSE techniques							
Mother	47	26.4	5	6.8	12.053 [1]	**<0.001**	0.219
Gynecologist	54	30.3	3	4.1	20.289 [1]	**<0.001**	0.284
Nurse/assistant	13	7.3	2	2.7	1.919 [1]	0.166	0.087
Health visitor	24	13.5	4	5.5	3.346 [1]	0.067	0.115
Self-taught	89	50.0	8	11.0	33.279 [1]	**<0.001**	0.364

Mean age: 23.93 ± 7.76. *Analyzed as multiple response. The bold values indicate statistically significant results where *p* < 0.05.

A total of 96.8% of students reported having heard about BSE and among them, 72.7% practiced it regularly. Additionally, 45.0% of respondents reported a positive family history of breast cancer, most frequently involving the grandmother, and this was significantly associated with a higher likelihood of performing BSE. This finding indicates that nearly one in two students had a close family member diagnosed with breast cancer. The most common sources of information on BSE were general practitioners (82.0%) and gynecologists (70.9%). Additionally, friends, acquaintances, and colleagues (76.4%), as well as family members (55.6%), played a significant role in disseminating.

information. Digital platforms also contributed to knowledge acquisition, with the internet (36.5%) and social media (25.8%) serving as notable sources. In contrast, schools and printed materials (e.g., flyers, posters) were less influential in providing BSE-related information. The majority of respondents (61.0%) reported having learned the correct technique for BSE independently. In addition, gynecologists (30.3%) and mothers (26.4%) played significant roles in teaching BSE. In contrast, healthcare professionals (e.g., nurses, assistants) and general practitioners contributed to a lesser extent in this process.

Statistical analyses showed a significant association between performing BSE and sources of information. Among those who performed BSE, 64.2% received information from a healthcare professional, while only 15.1% of those who did not perform BSE received such information. Information obtained from general practitioners also showed a significant correlation (82.0% vs. 9.6%). Information received from family members was also significantly associated with BSE practice (55.6% vs. 30.1%) particularly when BSE was learned from the mother (73.6% vs. 6.8%). Education provided by gynecologists (53.9% vs. 19.2%) and self-directed learning (50.0% vs. 11.0%) also showed a significant association with BSE practices.

Table 3 presents the association between BSE practice and dimensions of the HBM, including effect sizes (Cohen’s d) with corresponding 95% confidence intervals. Students who performed BSE were significantly older (*M* = 24.8 vs. 21.9 years, *p* = 0.007) and reported higher perceived susceptibility (*M* = 2.25 vs. 1.90, *p* = 0.003), seriousness (*M* = 2.95 vs. 2.70, *p* = 0.030), benefits (*M* = 4.03 vs. 3.60, *p* < 0.001), and self-efficacy (*M* = 3.05 vs. 2.15, *p* < 0.001), along with lower perceived barriers (*M* = 1.91 vs. 2.27, *p* < 0.001). No significant differences were observed in health motivation or perceived benefits of mammography, whereas perceived barriers to mammography were lower among BSE performers (*M* = 1.88 vs. 2.11, *p* = 0.036). Taken together, the analysis of HBM dimensions showed that self-efficacy emerged as the strongest predictor of BSE practice, indicating that confidence in one’s ability to correctly perform BSE is essential. Perceived barriers and lower perceptions of severity reduced the likelihood of practice, while better knowledge of BSE and trust in healthcare providers supported engagement.

**Table 3:** Association between breast self-examination practice and dimensions of the health belief model (HBM).

Variable	Performs BSE F (%) *N* = 178	Does not perform BSE *N* = 73	Total *N* = 251				
	M (SD)			*t*	*p*-value	Cohen’ *d*	95% CI (lower-upper)
Age	24.76 (8.40)	21.88 (5.30)	23.92 (7.77)	2.71	**0.007**	0.34	0.066–0.614
Susceptibility	2.25 (0.89)	1.90 (0.80)	2.15 (0.88)	2.96	**0.003**	0.41	0.135–0.685
Seriousness	2.95 (0.77)	2.70 (0.91)	2.87 (0.82)	2.18	**0.030**	0.30	0.026–0.574
BSE benefits	4.03 (0.75)	3.60 (0.96)	3.91 (0.84)	3.81	**< 0.001**	0.53	0.254–0.806
BSE barriers	1.91 (0.65)	2.27 (0.63)	2.01 (0.66)	−4.07	**< 0.001**	−0.57	−0.847 – −0.293
BSE self-efficacy	3.05 (0.75)	2.15 (0.84)	2.79 (0.87)	8.35	**< 0.001**	1.16	0.869–1.451
Health motivation	3.96 (0.59)	3.80 (0.60)	3.92 (0.59)	1.93	0.055	0.27	0.003–0.543
Mammography benefits	4.21 (0.89)	4.25 (0.96)	4.22 (0.91)	−0.34	0.737	−0.04	−0.312 – 0.232
Mammography barriers	1.88 (0.76)	2.11 (0.83)	1.94 (0.79)	−2.10	**0.036**	−0.29	−0.564 – 0.016

The bold values indicate statistically significant results where *p* < 0.05.

Following the bivariate analyses, a stepwise logistic regression analysis was conducted (Table 4). The independent variables included general characteristics and HBM dimensions that showed significant differences based on the t-test and chi-square test. The model was gradually expanded with relevant predictors and achieved its best fit at Step 7, where the −2 log-likelihood (−2LL) value was 132.045. The Omnibus test indicated a significant model fit, confirming that the model had a strong explanatory power. The final model explained 70.4% of the variance in BSE practice, demonstrating a robust predictive capacity.

**Table 4:** Logistic regression analysis for performing BSE.

Step	Variable	B	SE	Wald	*p*-value	OR	95% CI (lower-upper)
Step 1	BSE self-efficacy	−1.494	0.227	43.171	<0.001***	0.225	0.144–0.351
	Constant R^2^ Nagekerke = 0.309	2.989	0.582	26.363	<0.001***	19.861	–
Step 2	BSE learning: self-taught	−2.241	0.442	25.749	<0.001***	0.106	0.045–0.253
	BSE self-efficacy	−1.585	0.253	39.331	<0.001***	0.205	0.125–0.336
	Constant R^2^ Nagekerke = 0.453	3.889	0.691	31.665	<0.001***	48.838	-
Step 3	BSE learning: gynecologist	−2.536	0.655	14.982	<0.001***	0.079	0.022–0.286
	BSE learning: self-taught	−2.628	0.457	33.147	<0.001***	0.072	0.030–0.177
	BSE self-efficacy	−1.453	0.273	28.398	<0.001***	0.234	0.137–0.399
	Constant R^2^ Nagekerke = 0.538	4.055	0.759	28.554	<0.001***	57.664	–
Step 4	BSE learning: mother	−2.628	0.607	18.724	<0.001***	0.072	0.022–0.238
	BSE learning: gynecologist	−2.985	0.689	18.763	<0.001***	0.051	0.013–0.195
	BSE learning: self-taught	−3.324	0.508	42.856	<0.001***	0.036	0.013–0.097
	BSE self-efficacy	−1.487	0.308	23.281	<0.001***	0.226	0.124–0.414
	Constant R^2^ Nagekerke = 0.622	4.878	0.908	28.861	<0.001***	131.366	–
Step 5	BSE learning: mother	−2.822	0.637	19.607	<0.001***	0.059	0.017–0.207
	BSE learning: gynecologist	−3.332	0.744	20.064	<0.001***	0.036	0.008–0.154
	BSE learning: self-taught	−3.485	0.534	42.522	<0.001***	0.031	0.011–0.087
	Seriousness	−0.768	0.271	8.067	0.005**	0.464	0.273–0.788
	BSE self-efficacy	−1.543	0.320	23.275	<0.001***	0.214	0.114–0.400
	Constant R^2^ Nagekerke = 0.649	7.342	1.357	29.261	<0.001***	1543.111	–
Step 6	BSE learning: mother	−2.998	0.664	20.356	<0.001***	0.050	0.014–0.183
	BSE learning: gynecologist	−3.210	0.780	16.919	<0.001***	0.040	0.009–0.186
	BSE learning: self-taught	−3.581	0.562	40.599	<0.001***	0.028	0.009–0.084
	Seriousness	−1.129	0.311	13.188	<0.001***	0.323	0.176–0.595
	BSE barriers	1.176	0.387	9.243	0.002**	3.241	1.519–6.916
	BSE self-efficacy	−1.535	0.338	20.593	<0.001***	0.215	0.111–0.418
	Constant R^2^ Nagekerke = 0.680	5.851	1.458	16.097	<0.001***	347.491	–
Step 7	BSE learning: general practitioner	2.556	1.017	6.309	0.012*	12.881	1.753–94.626
	BSE learning: mother	−3.236	0.699	21.444	<0.001***	0.039	0.007–0.168
	BSE learning: gynecologist	−3.390	0.821	17.052	<0.001***	0.034	0.007–0.068
	BSE learning: self-taught	−3.856	0.597	41.681	<0.001***	0.021	0.172–0.606
	Seriousness	−1.130	0.321	12.404	<0.001***	0.323	1.408–6.802
	BSE barriers	1.130	0.402	7.903	0.005**	3.094	0.105–0.413
	BSE self-efficacy	−1.571	0.351	20.090	<0.001***	0.208	0.105–0.413
	Constant R^2^ Nagekerke = 0.704	3.815	1.694	5.070	0.024*	45.379	–

Significance *p < 0.05, **p < 0.01, ***p < 0.001. B, coefficient B; OR, odds ratio; SE, standard error; CI, confidence interval. The bold values indicate statistically significant results where *p* < 0.05.

In Step 7, several methods of acquiring self-examination skills were significant predictors. Learning from the mother, receiving information from a gynecologist, and self-learning were also significant in the model, although their odds ratios were below 1, suggesting that their predictive value was weaker when considered alongside other sources of information. In contrast, information obtained from a general practitioner showed the strongest positive association, increasing the likelihood of regular practice more than twelvefold.

According to the final model, self-efficacy was the most decisive predictor: students with higher self-efficacy were far more likely to perform regular BSE, whereas lower self-efficacy markedly reduced engagement. Perceived severity also played an important role, as lower perceptions of disease seriousness were linked to reduced BSE practice. Interestingly, perceived barriers were positively associated with BSE practice, a finding that may reflect overlapping effects between predictors or a greater awareness of challenges among students who already perform BSE.

Overall, the strongest predictors of BSE practice were self-efficacy, perceived severity, and information received from healthcare providers, particularly general practitioners. These findings highlight the importance of strengthening students’ confidence, addressing psychosocial factors, and ensuring credible professional guidance in promoting regular BSE.

Based on the results of the multivariate logistic regression presented in Table 5, information obtained from healthcare professionals significantly increases the likelihood of performing BSE. Women who received guidance from a gynecologist were 2.7 times more likely to perform self-examinations (OR = 2.673, *p* = 0.005), indicating a statistically significant association. Similarly, information provided by healthcare workers (e.g., nurses, assistants) increased the likelihood of performing self-examinations by 2.3 times (OR = 2.262, *p* = 0.032), also demonstrating a significant effect. The strongest effect was observed for information received from a general practitioner, as those who obtained guidance from their GP were more than four times more likely to perform self-examinations compared to those who did not receive such information (OR = 3.907). However, this association did not reach statistical significance (*p* = 0.077). Other sources of information, such as the media (TV) and family members, also increased the likelihood of performing self-examinations (OR = 3.231 and OR = 1.376, respectively). However, these results were not statistically significant (*p* = 0.075 and *p* = 0.315).

**Table 5:** The impact of information sources on breast self-examination—results of multivariate logistic regression.

Source of information	B (logit coefficient)	S.E.	Wald	*p*-value (Sig.)	Exp(B) (odds ratio)	95% CI lower-upper
Gynecologist	0.983	0.349	7.913	**0.005**	2.673	1.347–5.302
Healthcare professional	0.816	0.381	4.596	**0.032**	2.262	1.073–4.769
General practicioner	1.363	0.771	3.121	0.077	3.907	0.862–17.717
Family member	0.319	0.317	1.010	0.315	1.376	0.739–2.562
TV	1.173	0.658	3.179	0.075	3.231	0.890–11.730

## 4 Discussion

Breast self-examination (BSE) was originally recommended as a simple, cost-effective, and non-invasive method for the early detection of breast cancer. Research suggests that more than 90% of breast cancer cases can be detected by women themselves (38), further reinforcing the significance of BSE in early diagnosis and improving the chances of successful treatment. Despite its advantages, the effectiveness of BSE in reducing breast cancer mortality remains debated; therefore, many health organizations today emphasize its complementary role alongside clinical breast examinations and mammographic screening.

Although an organized breast cancer screening program is available in Hungary, participation rates are low, and women face numerous barriers that may hinder their attendance at screening appointments. In this context, regular and properly performed BSE continues to be an important tool for the early detection of breast cancer, particularly among those who do not participate in organized screening programs.

This study aimed to examine the knowledge, attitudes, and practices surrounding BSE among young women, while also identifying the psychological and sociodemographic predictors of regular practice. The HBM provided a theoretical framework for exploring how constructs such as perceived susceptibility, barriers, and self-efficacy influence BSE behavior. The research focused on female students of the Faculty of Health Sciences at the University of Pécs, as this population represents a critical stage in the development of preventive health behaviors, and has the potential to serve as a multiplier of health knowledge through future professional roles. Although routine BSE is not generally recommended for all young women, its assessment in this population remains highly relevant. Female health science students are at an age when preventive health behaviors are being formed, and they are also preparing for professional roles in which they will influence patients’ knowledge and attitudes. Their engagement with BSE may therefore have a dual impact: it contributes to their own health protection and positions them as credible role models in promoting breast cancer awareness and screening in wider society.

The findings of the present study revealed that BSE is influenced by a combination of psychological and educational factors. Among the constructs of the HBM, self-efficacy emerged as the most decisive predictor of BSE practice, while perceived barriers and perceived seriousness also played significant roles. Students who had acquired BSE knowledge through healthcare professionals, their mothers, or self-directed learning were more likely to engage in regular practice. Trust in healthcare providers and access to credible information sources further supported preventive behavior. Although general awareness of BSE was widespread in the sample, gaps remained in both the consistent application of BSE and accurate perception of personal risk.

In the current study, 96.4% of students reported having heard of BSE, a figure higher than that reported even among Syrian medical students (89.6%) (39, 40). Regarding practice, 70.9% of participants reported performing BSE, and 50.2% had done so within the past month. These rates are considerably higher than those found in Jordan (27.27%) (41), Turkey (13.32%) (42), Croatia (32.9%) (19), and Cyprus (10.9%) (43). Although awareness levels were generally high, a discrepancy remained between knowledge and consistent practice, indicating that awareness alone is not sufficient to ensure preventive behavior. Students who reported performing BSE showed significantly higher levels of perceived susceptibility than those who did not, particularly in cases where a family history of breast cancer, such as an affected grandmother, was reported. These results support the notion that a family history of the disease may not only serve as a medical risk factor but also enhance subjective risk perception, thereby encouraging regular BSE behavior (44, 45).

The majority of students perceived their own risk of breast cancer as low, which is consistent with findings from other studies (46, 47). This underestimation of personal risk may reduce motivation for regular BSE practice. In contrast, students with a higher sense of vulnerability were more likely to engage in self-examination. Although health motivation was assessed, it did not show a statistically significant association with BSE practice, differing from some prior findings (48). Overall, the results underscore the need to not only provide information but also improve individual risk perception and psychological readiness for action.

Compared to findings from international studies, the awareness and practice rates of BSE observed among Hungarian university students in the present study appear relatively high. While 96.4% of participants had heard of BSE and 70.9% reported performing it, the corresponding rates were notably lower in other countries: 27.27% in Jordan (41), 13.32% in Turkey (42), 32.9% in Croatia (19), and 10.9% in Cyprus (43). Even among Syrian medical students, the awareness rate was slightly lower (89.6%) than in our sample (39, 40). In Greece, although midwives practiced BSE more frequently than students, regular monthly practice remained uncommon (18). These findings suggest that Hungarian students show a relatively favorable alignment between awareness and practice, though the gap between knowledge and consistent behavior still exists.

In terms of HBM constructs, the results of the present study can be effectively compared to those of Turkish nursing students. The mean score for perceived susceptibility to breast cancer in our sample (*M* = 7.63) was similar to the values reported by Erbil and Bolukbas (7.56) (44), Özkan et al. (7.52) (49), and Yücel et al. (7.78) (50), but lower than that reported by Kılıç et al. (8.50) (51). Perceived seriousness was also at a high level (*M* = 21.65), closely aligned with findings from Erbil (22.44) (44) and Özkan (21.8) (49). The perceived benefits score in this study (*M* = 15.71) was slightly lower than that of Erbil (16.70) (44) and significantly lower than Yücel et al. (20.5) (50). Meanwhile, perceived barriers were somewhat higher (*M* = 24.02) compared to Erbil (22.96) (44) and Özkan (22.3) (49), and much higher than Kılıç et al. (16.29) (51), indicating that Hungarian students may perceive more obstacles to performing BSE.

Self-efficacy in the present study (*M* = 33.44) was comparable to scores reported by Erbil (36.23) (44) and Yücel (36.3) (50), but lower than that of Özkan (40.3) (49). The health motivation score (*M* = 25.42) was almost identical to those in Turkish samples, such as Özkan (26.6) (49) and Yücel (25.7) (50), but notably higher than in studies by Kılıç et al. (20.67) (51) and Gerçek et al. (19.25) (45). These cross-national comparisons highlight both cultural similarities and contextual differences in health beliefs and behaviors related to BSE. In particular, the higher perceived barriers and lower self-efficacy observed in the Hungarian context suggest a need for educational programs tailored to psychological empowerment and barrier reduction.

Consistent with the HBM, the present study confirmed that perceived benefits and self-efficacy are strong positive predictors of breast self-examination (BSE) practice, in line with previous findings (52, 53). Students with greater confidence in their ability to perform BSE were more likely to engage in the behavior, highlighting the crucial role of psychological empowerment. In contrast, perceived barriers were found to negatively influence BSE performance, echoing the conclusions of earlier research (29, 52–54). These barriers may include a lack of knowledge, uncertainty about proper technique, or emotional discomfort, all of which can discourage consistent practice.

Perceived susceptibility was also significantly associated with regular BSE, aligning with studies conducted in various international contexts (48, 55). However, this association was not identified as significant in a study among Saudi students (56), suggesting that cultural or contextual factors may influence whether perceived vulnerability translates into behavior. The findings regarding perceived seriousness also demonstrated a positive association with BSE in this study, although prior research has shown mixed results on this relationship (57, 58). These discrepancies may stem from differences in study design, population characteristics, or cultural norms. Indeed, studies from the United States and Indonesia have similarly shown that perceived seriousness can vary significantly by region and background (29, 47).

In addition to perceived seriousness, perceived benefits also emerged as a key factor in influencing BSE behavior. Interestingly, most participants in the present study reported low levels of perceived benefits, similar to results observed in certain African populations (59), while moderate benefit levels have been reported in Southeast Asia (29). These patterns support the idea that perceptions of benefit—like other HBM constructs—are shaped by cultural and educational contexts. Regarding barriers, participants in our study tended to report either low or high scores, with few reporting moderate levels, consistent with findings from Taiwan and Indonesia (29, 54). Moreover, higher barriers were associated with lower BSE practice, in agreement with studies from Oman (60) and Saudi Arabia (56), while research among Nigerian students found no such significant relationship (55), again pointing to possible cultural or contextual variability.

Self-efficacy emerged as one of the strongest predictors of BSE, reinforcing results from previous studies conducted in Iran (61), Oman (62), and Ethiopia (59). In particular, research among Iranian university students revealed that women with high self-efficacy were up to 13 times more likely to engage in BSE (61, 62), further underlining the critical influence of confidence and psychological readiness. Although health motivation was included in the present analysis, no statistically significant association was observed with BSE performance, which diverges from certain prior studies (48). This result may indicate that while motivation is an important background factor, it does not necessarily lead to action unless combined with other enabling beliefs, such as self-efficacy and perceived benefits.

Altogether, these findings suggest that promoting BSE behavior requires more than disseminating information. Effective interventions must enhance perceived susceptibility, reduce perceived barriers, and most importantly, build women’s confidence in their ability to carry out BSE. The results also reinforce the importance of interpersonal sources, such as healthcare professionals, general practitioners, and family members, in shaping preventive behavior. These trusted individuals not only provide accessible and credible information but also offer emotional and practical support that may be especially influential among young adults. Such findings support extending the traditional HBM to include external social influences, which are often crucial in translating knowledge into action.

The findings of the present study support the applicability of the HBM in understanding and promoting regular breast self-examination (BSE) behavior among young women. In particular, the strong predictive role of self-efficacy highlights the need for public health interventions that go beyond information provision and focus on psychological empowerment. Greater confidence in one’s ability to perform BSE was consistently associated with regular practice, reinforcing the conclusion that self-efficacy may be more influential than awareness alone (63, 64).

Given that the majority of students perceived their own breast cancer risk as low despite high awareness levels, interventions should address this false sense of security by enhancing personal risk perception. The development of health education strategies that reinforce the perceived benefits of BSE, while simultaneously reducing perceived barriers, is essential for translating awareness into action. This is particularly important among university students, as this stage of life presents a key opportunity to influence long-term preventive behaviors.

Educational institutions have an important role in supporting this goal. Universities could incorporate BSE-related content into health curricula, provide hands-on training using breast models, and offer accessible digital learning tools, such as instructional videos or mobile apps. Furthermore, peer-led workshops and follow-up sessions could foster a supportive environment for practicing BSE and improving self-efficacy. Involving trusted individuals, such as healthcare professionals, general practitioners, and family members, may also enhance motivation and reinforce positive attitudes.

Overall, the results justify the development of targeted educational and communication strategies that help young women develop the necessary skills, motivation, and confidence to engage in regular BSE. These strategies can ultimately contribute to earlier detection, improved outcomes, and a greater culture of health consciousness within the population. The findings also underscore the relevance of integrating both psychological and social dimensions into public health planning, further extending the practical value of the HBM framework in designing effective health promotion programs. Empowering young women with the skills and confidence to perform regular BSE is not merely a preventive strategy; it is an investment in the future of women’s health.

## 5 Strengths and limitations

The present research provides valuable insight into the health beliefs and breast self-examination (BSE) practices of female university students in Hungary, a population that has been underrepresented in previous literature. Conducted prior to an educational intervention, the study allowed for an accurate assessment of baseline knowledge and beliefs. A Hungarian translation of the Champion Health Belief Model Scale (CHBMS), reviewed by experts and tested in a pilot study, was used to support the reliability and clarity of the questionnaire. Additionally, the 100% response rate and inclusion of students from various academic disciplines strengthen the representativeness and internal validity of the findings. These results can inform the development of future public health education strategies and university curricula aimed at improving breast cancer prevention awareness among young women.

This study has several limitations that should be considered when interpreting the findings. First, one of the key limitations of this study is its reliance on a non-random, convenience sample drawn from a single university faculty, consisting exclusively of young female health sciences students. This group may possess higher health literacy and greater awareness of preventive behaviors compared to the general population, which limits the generalizability of the findings to broader populations, age groups, and educational settings. Therefore, the findings should be interpreted as exploratory and not representative of all young women in Hungary. Second, data were collected through self-reported questionnaires without objective verification of knowledge or behavior, particularly regarding self-reported breast self-examination (BSE) practice. This approach may introduce recall and social desirability bias, as participants might overreport positive health behaviors or underestimate perceived barriers. Third, the cross-sectional design of the study precludes causal interpretations. The associations observed between health beliefs and BSE practice cannot confirm cause-and-effect relationships. Fourth, the study did not include qualitative methods that could have provided deeper insights into cultural, emotional, or attitudinal factors influencing women’s health beliefs and preventive behaviors. Future research incorporating qualitative interviews or focus groups could help explore these dimensions in greater depth. Despite these limitations, the study provides meaningful insights into the health beliefs and preventive behaviors of female health science students, a group of particular importance as future health professionals. These findings may serve as a basis for developing evidence-based, student-centered health education programs and underline the need for future multicenter, randomized studies with larger and more diverse samples.

## 6 Conclusion

The study results indicate that BSE practice is primarily influenced by self-efficacy, perceived barriers, perceived severity, and the methods used to learn self-examination. High self-efficacy significantly increases the likelihood of performing BSE, whereas perceived barriers, such as lack of knowledge or fear of incorrect technique, reduce it. The study also highlights the importance of personal learning sources: students who learned the correct technique independently, from their mother, or from a gynecologist were more likely to perform regular self-examinations. Low perception of disease severity also negatively affected BSE practice, emphasizing the importance of educational programs in raising awareness of breast cancer risks. The findings suggest that healthcare professionals, particularly general practitioners and gynecologists, play a key role in providing accurate information and educating individuals on self-examination techniques. This study holds particular significance as it was conducted among students in health sciences higher education, who will not only shape their own health behaviors but, through their professional roles, directly influence the health awareness of their communities and future patients. With the right knowledge and a positive attitude, they can contribute to improving public health behaviors, particularly in breast cancer prevention, raising awareness of the importance of mammographic screenings, and encouraging regular breast self-examinations.

These findings underline the importance of structured BSE education tailored to young women in academic settings. By addressing cognitive factors such as perceived benefits, barriers, and self-efficacy, and by utilizing engaging, practical formats, universities can play a key role in promoting early detection behaviors among future generations. When developing prevention strategies, it is crucial to focus on programs that enhance self-efficacy and reduce perceived barriers. Targeted educational interventions can contribute to improving the early detection of breast cancer, thereby reducing morbidity and mortality associated with the disease (65, 66). Further research is needed to assess the effectiveness of educational methods and to develop interventions that promote sustainable, long-term health-conscious behaviors.

At the same time, it should be emphasized that these findings should be interpreted with caution when generalizing to the entire population due to the single-site, non-random sample. Nevertheless, they provide important insights into the health behaviors of young women and future healthcare professionals, highlighting a subgroup that plays a crucial role in shaping both personal and community health practices.

Future research should focus on conducting multicenter studies with randomized sampling to ensure broader representativeness and to strengthen the reliability, validity, and generalizability of the present results in more diverse populations. Such studies would enhance the evidence base for developing large-scale, effective educational interventions aimed at improving breast cancer prevention behaviors among women. Furthermore, qualitative research exploring cultural and attitudinal factors could provide deeper insights into the underlying beliefs, motivations, and contextual influences shaping women’s preventive health behaviors. In addition, healthcare professionals should place greater emphasis on the continuous promotion and teaching of breast self-examination to ensure that awareness is not only raised but also translated into sustainable preventive practices.

## Data Availability

The raw data supporting the conclusions of this article will be made available by the authors, without undue reservation.
